# Imaging of plantar fascia disorders: findings on plain radiography, ultrasound and magnetic resonance imaging

**DOI:** 10.1007/s13244-016-0533-2

**Published:** 2016-12-12

**Authors:** Ferdinando Draghi, Salvatore Gitto, Chandra Bortolotto, Anna Guja Draghi, Gioia Ori Belometti

**Affiliations:** 1 Radiology Department, Fondazione IRCCS Policlinico San Matteo, Università degli Studi di Pavia, Pavia, Italy; 2 Postgraduation School in Radiodiagnostics, Università degli Studi di Milano, Via Festa del Perdono 7, 20122 Milano, Italy

**Keywords:** Plantar fascia, Fasciitis, Fibromatosis, Tear, Imaging

## Abstract

Plantar fascia (PF) disorders commonly cause heel pain and disability in the general population. Imaging is often required to confirm diagnosis. This review article aims to provide simple and systematic guidelines for imaging assessment of PF disease, focussing on key findings detectable on plain radiography, ultrasound and magnetic resonance imaging (MRI). Sonographic characteristics of plantar fasciitis include PF thickening, loss of fibrillar structure, perifascial collections, calcifications and hyperaemia on Doppler imaging. Thickening and signal changes in the PF as well as oedema of adjacent soft tissues and bone marrow can be assessed on MRI. Radiographic findings of plantar fasciitis include PF thickening, cortical irregularities and abnormalities in the fat pad located deep below the PF. Plantar fibromatosis appears as well-demarcated, nodular thickenings that are iso-hypoechoic on ultrasound and show low-signal intensity on MRI. PF tears present with partial or complete fibre interruption on both ultrasound and MRI. Imaging description of further PF disorders, including xanthoma, diabetic fascial disease, foreign-body reactions and plantar infections, is detailed in the main text. Ultrasound and MRI should be considered as first- and second-line modalities for assessment of PF disorders, respectively. Indirect findings of PF disease can be ruled out on plain radiography.

*Teaching Points*

• *PF disorders commonly cause heel pain and disability in the general population*.

• *Imaging is often required to confirm diagnosis or reveal concomitant injuries*.

• *Ultrasound and MRI respectively represent the first- and second-line modalities for diagnosis*.

• *Indirect findings of PF disease can be ruled out on plain radiography*.

## Introduction

Plantar fascia (PF) disorders are common in the adult population [1]. They cause pain and disability and may curtail the performance of athletic activities, work-related duties or routine tasks [2]. Imaging is of great help for achieving correct diagnosis, prompting appropriate treatment and aiding in the determination of prognosis. Awareness of the normal and pathological imaging appearance of the PF is thus required. This review article aims to provide radiologists and clinicians with simple and systematic guidelines for the evaluation of PF disorders, specifically focussing on key features suggestive of PF disease that have to be detected on conventional radiograph, ultrasound and magnetic resonance imaging (MRI). These guidelines are generated from our centres’ experience in combination, as indicated by the references in the text, with a thorough analysis of the last 20 years’ literature (1996–2016). A systematic search of the literature was carried out in PubMed using the keywords “plantar fascia” or “plantar aponeurosis” combined with “radiography”, “X-ray”, “ultrasound”, “sonography”, “magnetic resonance imaging” or “imaging”, even combined with “fasciitis”, “fibromatosis”, “tear”, “rupture”, “xanthoma”, “diabetes mellitus”, “infection” or “foreign body”. Additionally, the references of identified publications were checked. Original studies and review articles in English dealing with imaging description of PF and related disorders were included. Case reports and case series were selected according to clinical relevance.

## Anatomy and function of the PF

**Fig. 1:**
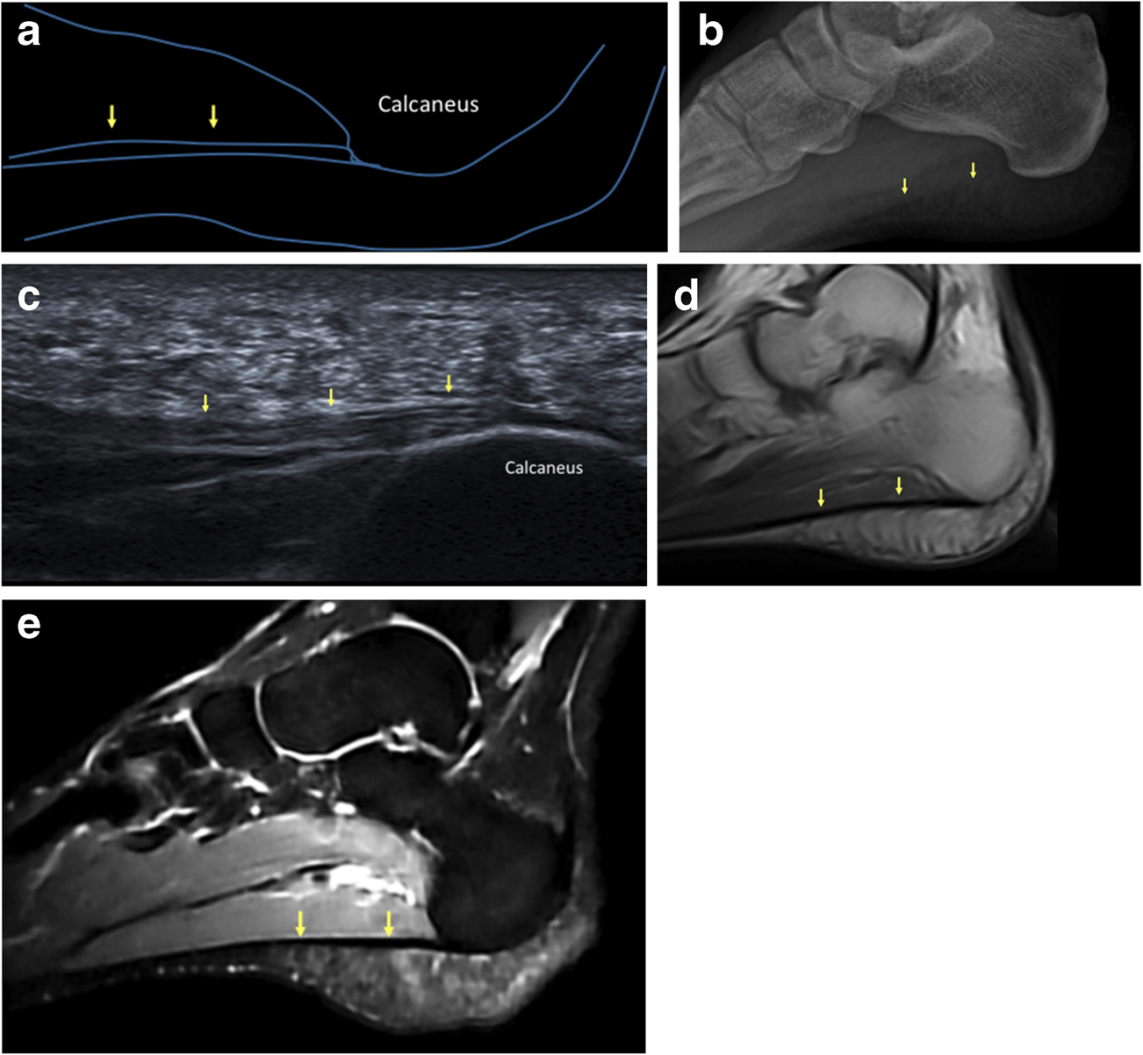
Normal plantar fascia. A schematic representation (**a**) and lateral plain radiograph (**b**) show the normal PF (*arrows*). On sagittal ultrasound scan, the normal PF (*arrows*) appears as a fibrillar ligamentous structure (**c**). On MRI, the normal PF (*arrows*) is seen as a thin band of low signal intensity on both T1-weighted (**d**) and fluid-sensitive (**e**) images

The PF (Fig. 1), also called the plantar aponeurosis, is a strong connective tissue structure that helps maintain the longitudinal arch of the foot [3, 4]. The PF consists of three bundles: central, lateral and medial. The central component is proximally thick and distally thin and is the thickest of the three. It arises from the medial tubercle of the calcaneus and extends distally becoming broader and covering the plantar surface of the flexor digitorum brevis muscle. Distally, it divides into five digitations that insert into the metatarsophalangeal joints. The lateral portion is also proximally thick and distally thin. It arises from the lateral margin of the medial calcaneal tubercle, covers the plantar surface of the abductor digiti minimi muscle and inserts into the fifth metatarsal joint capsule. The medial portion is thinner than the others. It arises from the midportion of the central bundle, covers the plantar surface of the abductor hallucis muscle and inserts into the first metatarsal joint capsule [5]. The mean maximal thickness of the PF has been reported as 4.0 mm in its central bundle, 2.3 mm in its lateral bundle and 0.6 mm in its medial bundle [6]. Overall, PF thickness is greater in men than in women [6]. Histologically, the PF is mostly composed of type I collagen fibres forming bundles arranged in a proximal-distal direction, with a few transverse and vertical collagen fibres. These large fibrous bundles are embedded within a matrix of loose connective tissue containing type III collagen and a few elastic fibres [5].

## General features of PF disorders

**Fig. 2:**
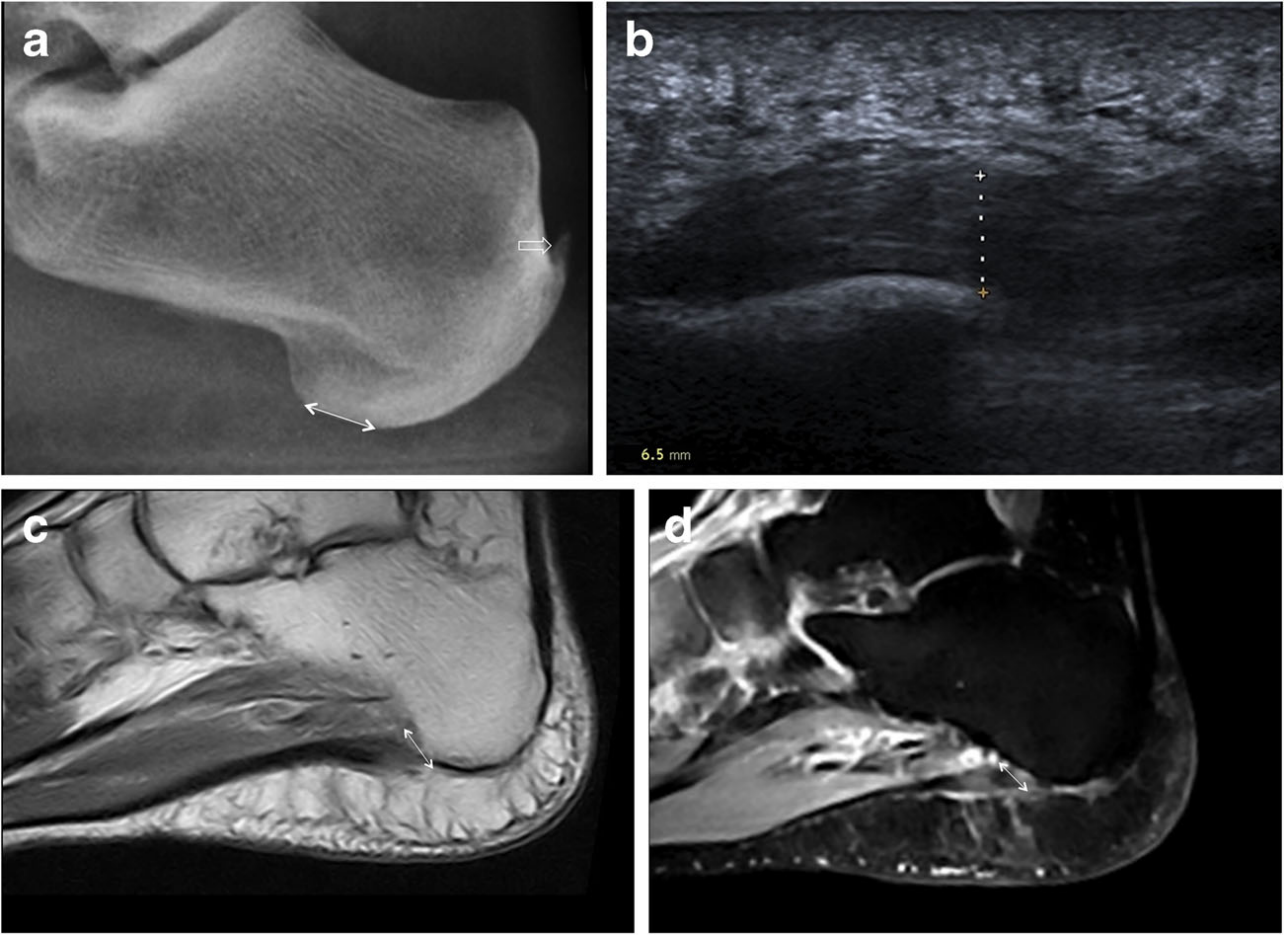
Plantar fasciitis. Lateral plain radiograph highlights an increase in the distance between subcutaneous fat and intrinsic muscles of the foot at the calcaneal insertion of the PF as an indirect sign of plantar fasciitis (*double-head arrow*); calcific enthesopathy of the Achilles tendon is also seen (*open arrow*) (**a**). On ultrasound, plantar fasciitis presents with PF thickening (*dashed line*, 6.5 mm), a hypoechoic appearance and loss of fibrillar pattern (**b**). MRI confirms thickening of the PF at its calcaneal origin (*double-head arrow*) with intrasubstance areas of intermediate and high signal intensity on T1-weighted (**c**) and fluid-sensitive (**d**) images, respectively

**Fig. 3:**
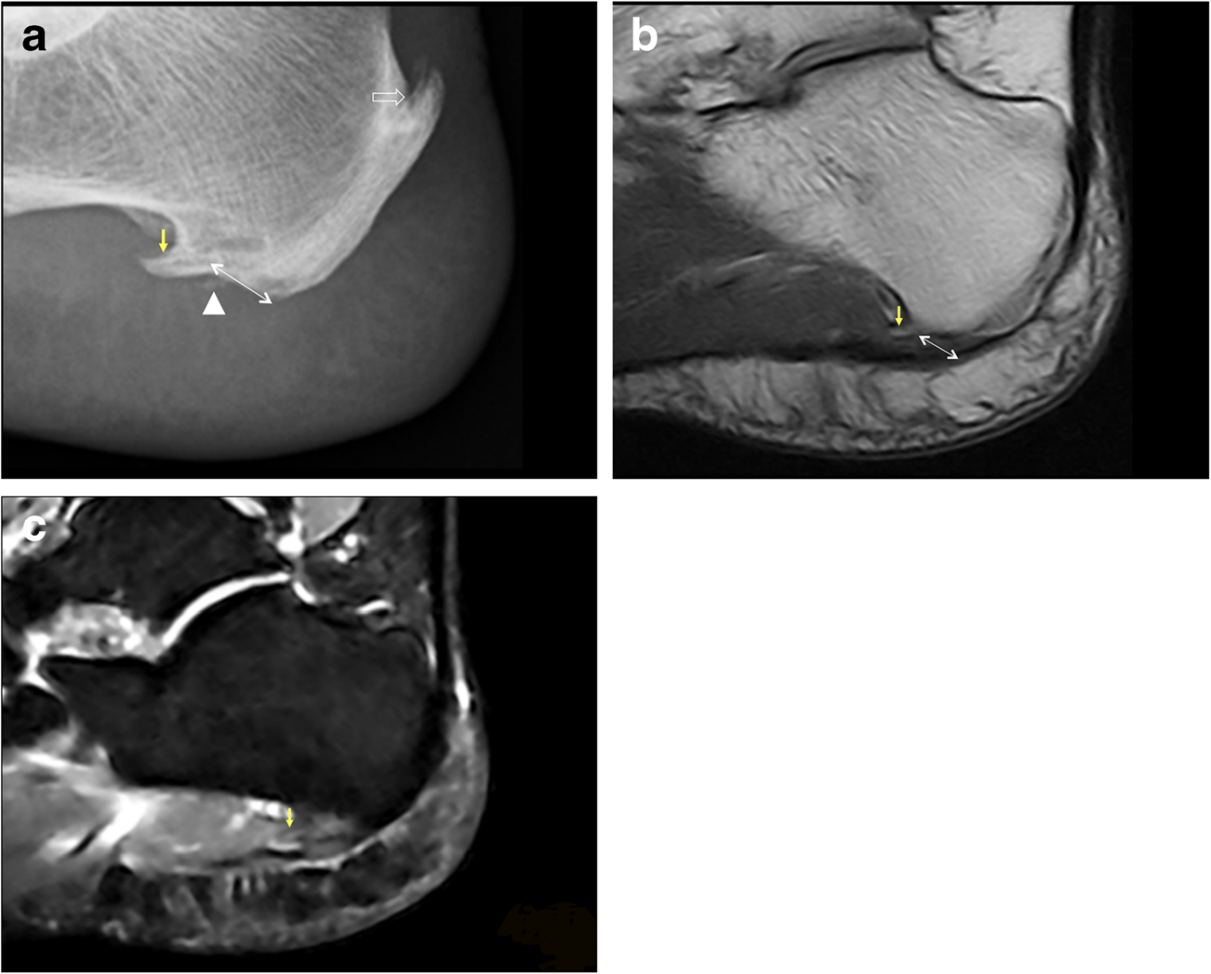
Plantar fasciitis. Lateral plain radiograph shows PF thickening (*double-head arrow*) and fine calcifications at the calcaneal insertion of the PF (*arrowhead*); a plantar calcaneal spur at the origin of intrinsic muscles of the foot (*arrow*) and calcific enthesopathy of the Achilles tendon (*open arrow*) are also evident (**a**). MRI confirms the presence of a calcaneal spur (*arrow*) and PF thickening at its calcaneal attachment (*double-head arrow*) (**b**). Bone marrow oedema in the calcaneal spur (*arrow*) is demonstrated on the fluid-sensitive image (**c**)

**Fig. 4:**
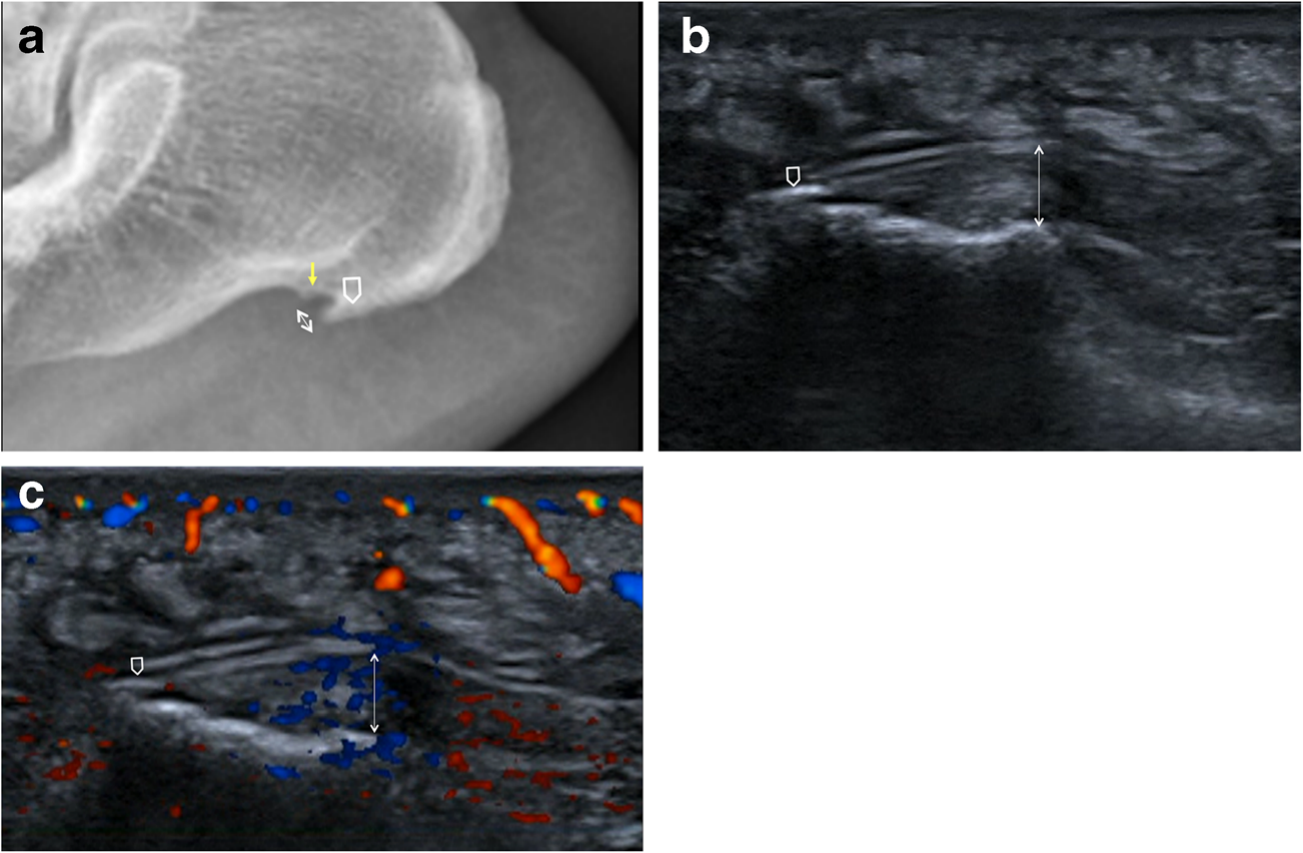
Plantar fasciitis. Lateral plain radiograph shows PF thickening (*double-head arrow*), a calcaneal spur located within the PF (*open arrow*) and another spur at the origin of intrinsic muscles of the foot (*arrow*); cortical irregularities of the calcaneus with sclerotic changes are also seen (**a**). On ultrasound, the PF is thickened and hypoechoic (*double-head arrow*) with a minute calcification (*open arrow*) at its insertion into the calcaneus (**b**). On colour-Doppler ultrasound, hypervascularisation of the PF and adjacent soft tissues is demonstrated (**c**)

Plantar fasciitis (Figs. 2, 3, and 4) is the most common injury of the PF and is estimated to induce more than 1 million patients to seek treatment annually [7]. Despite its name, plantar fasciitis has a degenerative rather than inflammatory nature and is related to overuse trauma leading to microtears [8]; thus, the term “plantar fasciopathy” is often preferred. The proximal third of the central bundle of the PF is classically involved; however, distal plantar fasciitis has recently been recognised as a cause of recalcitrant heel pain [9]. The aetiology of plantar fasciitis is multifactorial. Biomechanical risk factors include those causing repetitive stress on the PF, such as foot deformities, improper footwear, increased body mass index and activities that involve prolonged walking, running or standing [10–12]. Among the medical conditions associated with plantar fasciitis, the most notable are seronegative spondyloarthropathies and rheumatoid arthritis [13–16]. Plantar calcaneal spurs, also known as calcaneal enthesophytes, have been investigated in great detail as a possible cause of plantar fasciitis [17–20], but they are not specific and also occur in asymptomatic individuals. The diagnosis of plantar fasciitis generally relies on clinical history and physical examination. The main symptoms include pain and stiffness in the morning, or pain at the beginning of activity after rest. Physical examination reveals tenderness at the origin of the PF and impaired dorsiflexion of the ankle and extension of the toes [21–24]. Although generally self-limiting, plantar fasciitis may result in physical inactivity and impact quality of life. Imaging can aid in the diagnosis, particularly in recalcitrant cases or may rule out other heel pathology [9, 22].

**Fig. 5:**
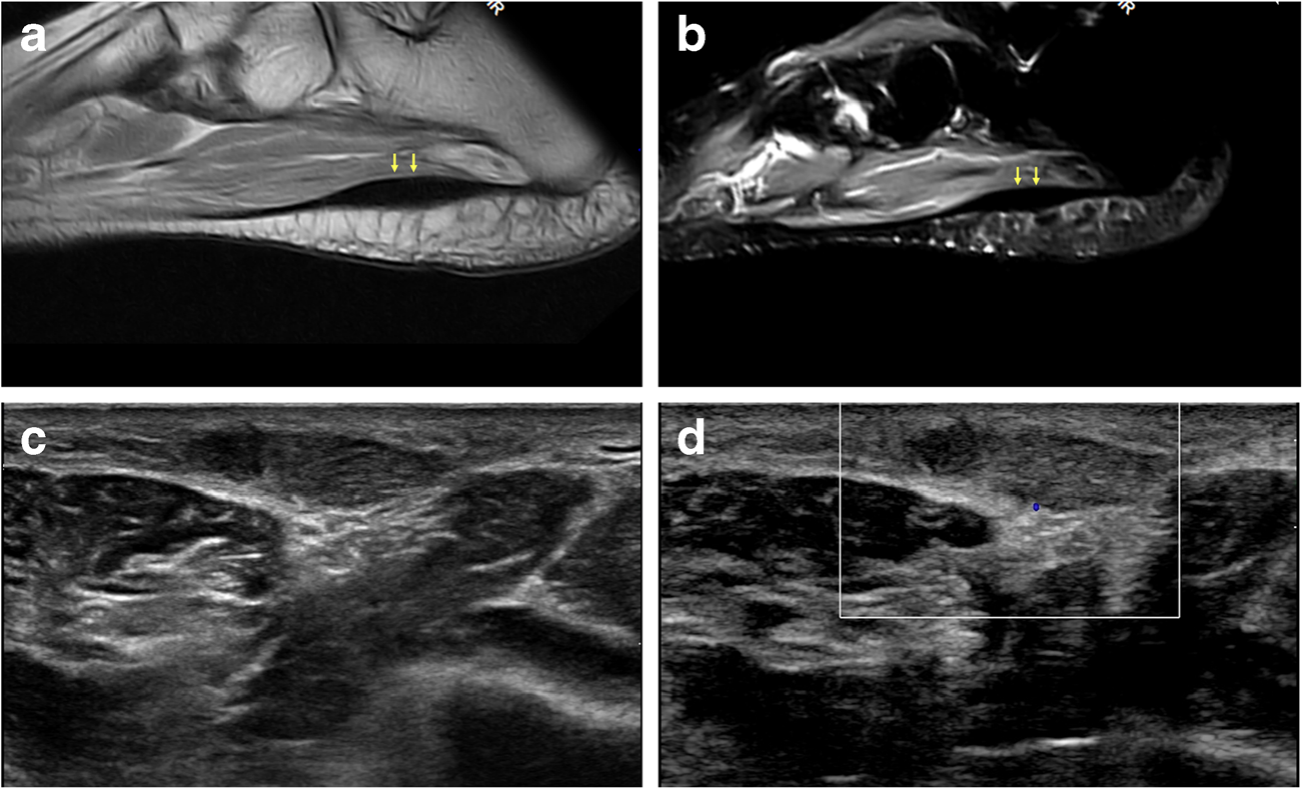
Ledderhose disease. MRI shows a fusiform thickening (*arrows*) in the distal portion of the PF with low signal intensity on both T1-weighted (**a**) and fluid-sensitive images (**b**). On ultrasound, a well-demarcated, hypoechoic nodule is demonstrated (**c**) with no increased internal vascularity (**d**)

Plantar fibromatosis or Ledderhose disease (Fig. 5) is a benign nodular formation due to fibroblastic proliferation in the PF. It tends to involve the distal two thirds of the PF, usually in its central bundle, although proximal nodules are not uncommon. Nodular lesions may be multiple and bilateral and typically measure less than 3 cm in diameter [25–27]. Plantar fibromatosis is frequently seen as an isolated disease, but an association with Dupuytren’s disease has been noted [28]. Clinically, plantar fibroma appears as a firm thickening or a single nodule, generally localised in the medial portion of the sole, which is occasionally painful [25–27].

**Fig. 6:**
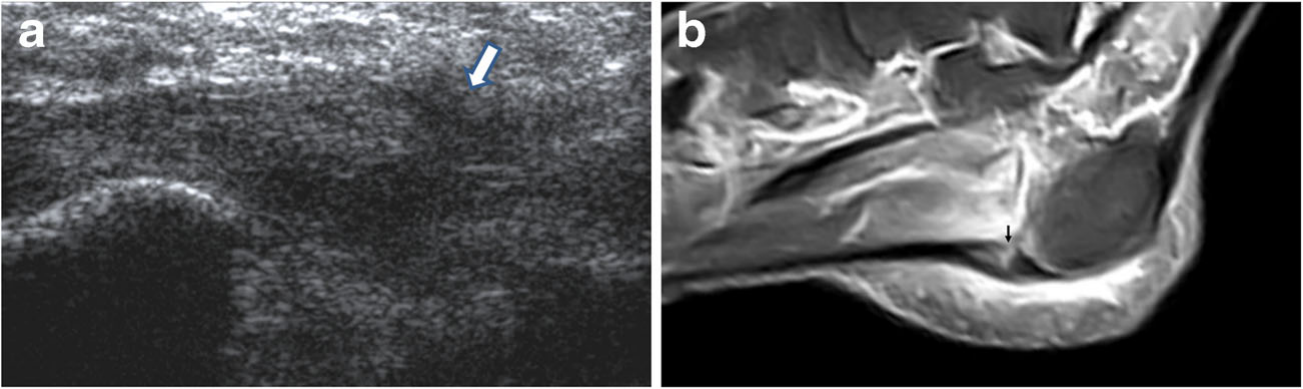
Plantar fascia rupture. On ultrasound, a tear in the PF (*arrow*) is shown; the PF is hypoechoic and thickened as a result of previous plantar fasciitis treated with local injections (**a**). MRI confirms PF rupture (*arrow*) and highlights marked oedema of soft tissues (**b**)

Tears of the PF (Fig. 6) are uncommon and can be partial or complete. Traumatic tears are often related to forcible plantar flexion of the foot in competitive athletes, mostly runners and jumpers; these are typically distal to calcaneal insertion of the PF and chronic overuse is considered an aetiological factor [29–32]. Spontaneous ruptures may occur at the calcaneal attachment of the PF in patients with previous history of plantar fasciitis and local treatment with steroid injections [33–35]. Clinical presentation includes acute pain, usually accompanied by a “snap” noise, and local swelling [36].

**Fig. 7:**
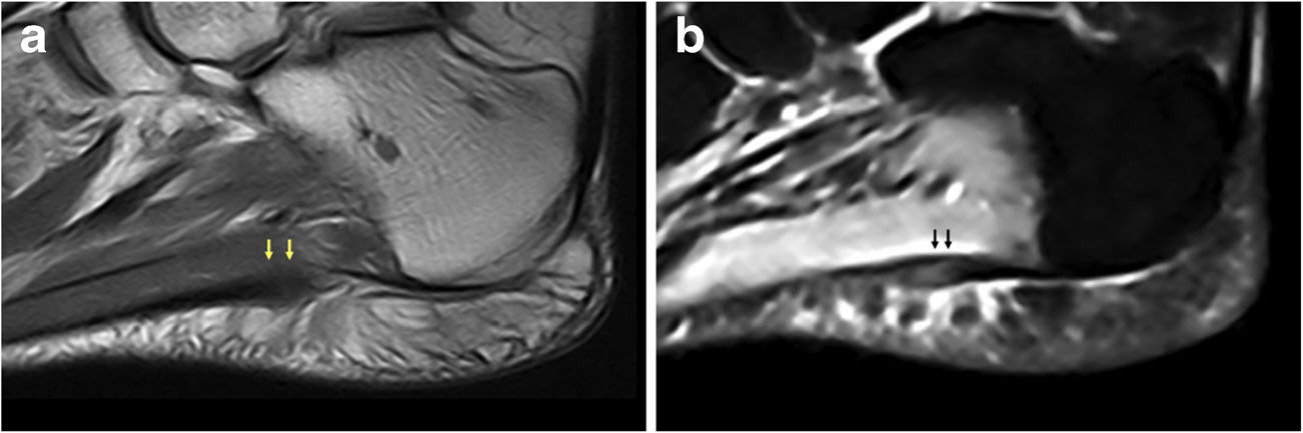
Plantar xanthoma. On both sagittal T1-weighted (**a**) and fluid-sensitive (**b**) images, xanthoma (*arrows*) appears as fusiform enlargement of the PF and shows heterogeneous signal intensity

**Fig. 8:**
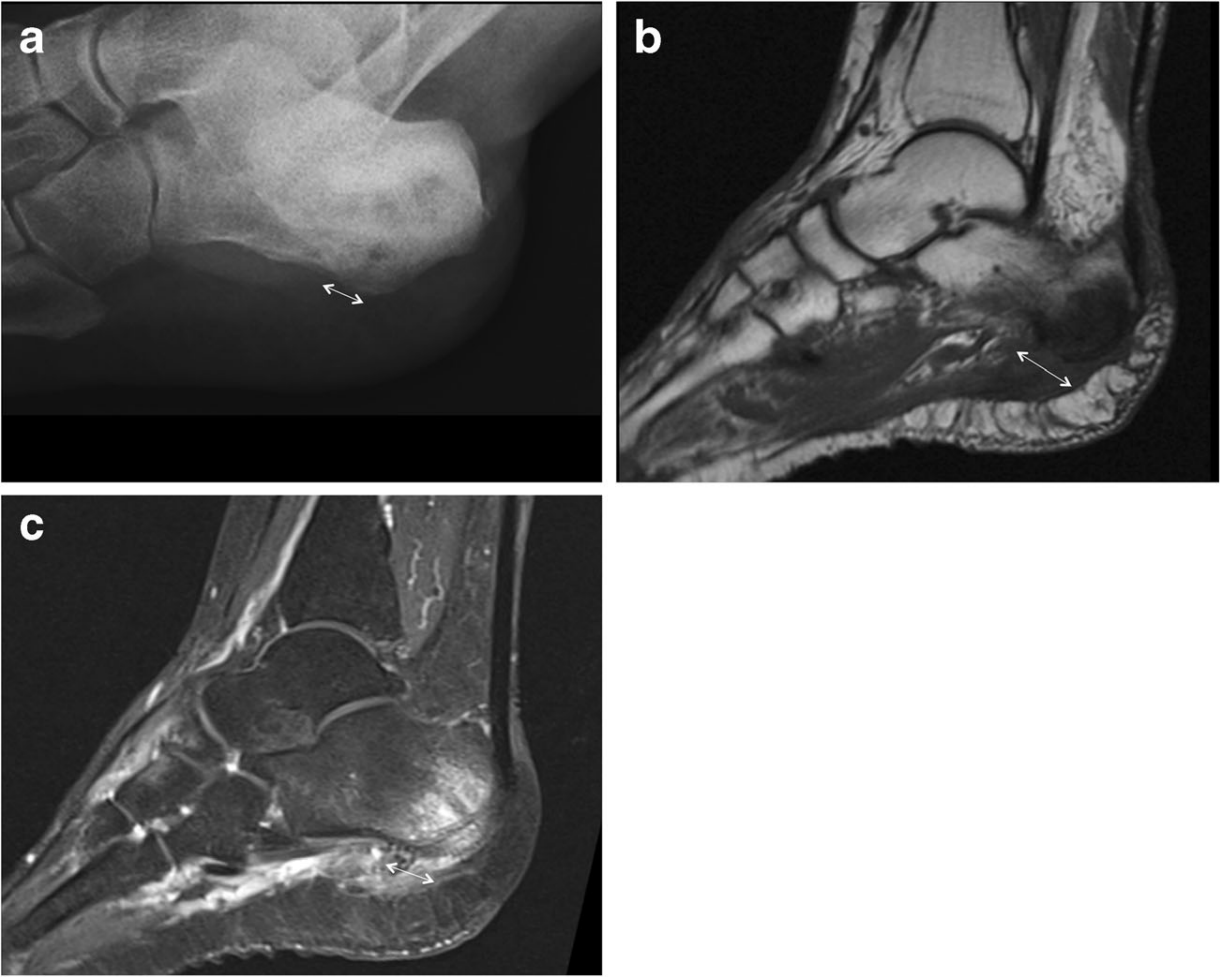
Heel osteomyelitis. Lateral plain radiograph shows marked morphological alteration of the heel with irregular lytic areas and concomitant PF thickening (*double-head arrow*) due to spreading of the infection (**a**). MRI confirms morphological alterations of the heel and PF (*double-head arrow*) on both T1-weighted (**b**) and fluid-sensitive (**c**) images

Peculiar lesions of the PF should be kept in mind as differential diagnoses of the main PF disorders and include xanthoma, diabetic fascial disease, foreign-body reactions and plantar infections. Xanthomas (Fig. 7) are described in many hyperlipidaemia states; they typically involve tendons and are occasionally located within the PF. They are usually asymptomatic and tend to recur after surgical removal [3]. Some studies have found that the thickness and stiffness of the PF and Achilles tendon are increased in patients with type I and type II diabetes mellitus [37, 38]. PF thickness is considered a predictor of the development of late complications in type I diabetes mellitus [38, 39]. Moreover, a relationship between PF thickening in type II diabetes mellitus and body mass index values has also been demonstrated [37]. Occasionally, foreign material is present within or adjacent to the PF and presents with symptoms of plantar fasciitis. It derives from penetrating injuries even though history of trauma or puncture is not always reported [3]. Infectious fasciitis (Fig. 8) may occur as a result of spread from a contiguous source of infection, penetrating wounds due to iatrogenic (surgical procedures) and accidental causes (foreign body, punctures), or in diabetics’ feet [2, 40]; atypical infections may result from haematic diffusion of microorganisms, particularly in immunosuppressed patients [41]. As fascial inflammation can cause destruction of mechanical barriers, infection may spread and affect perifascial structures including soft tissues, bone and muscles [42].

## Plain radiography

Plain radiography is widespread, cost-effective and panoramic, thus often representing the imaging modality of first choice for the evaluation of painful heel.

Several findings suggestive of plantar fasciitis can be detected on conventional radiographs. Despite this, plain radiography should not be used to make a diagnosis of plantar fasciitis without knowledge of clinical history or physical examination findings [19]. PF thickness can be measured with accuracy on lateral plain radiographs of the ankle and foot [3, 4, 19]. PF mean thickness at its calcaneal origin, in the central fascicle, is 4.0 mm [6]. Increased thickness of the PF measuring more than 4–5 mm within 5 mm of its calcaneal attachment is evident on lateral plain radiographs of individuals with plantar fasciitis (Figs. 2, 3 and 4) and represents a reliable sign of plantar fasciitis [19]. Deep below the PF, at its calcaneal origin, a fat pad is seen and usually has a triangular shape on lateral non-weight-bearing plain radiographs. This fat pad becomes narrowed or is absent in individuals with plantar fasciitis, maybe owing to both mechanical and inflammatory mechanisms, thus representing a further sign of plantar fasciitis [19]. The combination of thickened PF and fat pad abnormalities on lateral plain radiography has a sensitivity of 85% and specificity of 95% for plantar fasciitis [19]. Changes in the cortex of the calcaneus at the attachment of the PF, with or without spur formation, have been correlated with plantar fasciitis (Fig. 4). These are cortical irregularities presenting with loss of the smooth contour of the bone and both cortical lucency and sclerosis [19].

Plantar calcaneal spurs and calcifications within the PF are uncommon occurrences in patients with plantar fasciitis [19, 20]. The significance of calcaneal spurs as a cause of plantar fasciitis has received considerable attention in the literature [17–20], and currently their importance in terms of the diagnosis and prognosis of plantar fasciitis is debatable. Calcaneal spurs associated with plantar fasciitis include those located within the plantar fascia (Fig. 4) [20]. These are however very uncommon, as the most common site of plantar calcaneal spurs is in the abductor hallucis and flexor digitorum brevis origins, deep below the PF (Figs. 3 and 4) [18, 20]. Thus, evidence of calcaneal spurs on conventional radiographs is not a pathognomonic sign of plantar fasciitis.

Apart from highlighting indirect signs of plantar fasciitis and calcaneal spurs, conventional radiography is useful in overviewing anatomical and pathological changes of the bone and soft tissues. Radiopaque foreign material, such as metals, may be easily revealed [43]. In the case of infectious fasciitis, plain radiography shows soft-tissue swelling and blurring of soft-tissue planes. Concomitant osteomyelitic changes in bone morphology can also be detected and mainly include lytic lesions, osteopenia, loss of trabecular architecture, new bone apposition and periosteal thickening (Fig. 8) [40]. Finally, stress fractures may be associated with PF injuries and should be ruled out [44].

## Ultrasound

On ultrasound, similarly to ligaments, PF shows a fibrillar pattern due to the hyperechoic appearance of type I collagen fibre bundles embedded within a background of hypoechoic matrix [45, 46].

Sonographic characteristics of plantar fasciitis include loss of fibrillar structure, increased thickness over 4 mm, perifascial collections and calcifications within the PF (Figs. 2 and 4) [16, 47–57]. Hyperaemia is a well-known feature of tendinopathy due to neurovascular growth and may contribute to pain. It can be assessed using Doppler ultrasound. Similarly, Doppler ultrasound can identify hyperaemia in the PF, near its proximal insertion and in the perifascial soft tissue, in patients with plantar fasciitis (Fig. 4); hyperaemia can also correlate with treatment [58–61]. As an additional finding, in patients with plantar fasciitis the PF is less elastic under real-time sonoelastography, and this might increase the accuracy of ultrasound [62, 63].

The sonographic presentation of plantar fibromatosis (Ledderhose disease) includes typically single, rarely multiple iso-hypoechoic, well-demarcated, nodular thickenings of the PF, with no calcifications or fluid collection. Doppler ultrasound generally shows no vascular flow inside the lesion (Fig. 5) [64].

Sonographic findings of PF rupture include complete or partial interruption of the PF, with hypoechoic tissue at the site of rupture related to local haemorrhage and inflammation (Fig. 6) [3].

Among other disorders of the PF, plantar xanthomas appear as nodules with a speckled pattern [3]. Increased thickness of the PF can be sonographically detected in the early stages of diabetes mellitus [37–39]. Ultrasound can aid in the diagnosis of foreign body reaction by identifying echoic extraneous material within or adjacent to the PF. Sometimes posterior acoustic shadowing and, in cases of metal objects, comet tail reverberation artefacts may also be seen [65]. Ultrasound is useful in the assessment of musculoskeletal infections, particularly in distinguishing acute or chronic infections from tumours or non-infective conditions. In the case of infectious fasciitis, the PF is increased in volume with loss of fibrillar pattern and perifascial oedema and is hyperaemic on Doppler evaluation [40].

## Magnetic resonance imaging

In healthy individuals, the PF is homogeneously hypointense on both T1-weighted and fluid-sensitive sequences [6].

MRI findings of plantar fasciitis include: thickening of the PF, most commonly at its calcaneal origin; intrasubstance areas of intermediate signal on T1-weighted sequences and increased signal on fluid-sensitive sequences; oedema in the adjacent soft tissue; bone marrow oedema of the calcaneal attachment of the PF suggestive of enthesopathy (Figs. 2 and 3) [2, 4].

In plantar fibromatosis (Ledderhose disease), the common MRI appearance of plantar fibroma is a lobulated mass of low signal intensity on both T1- and T2-weighted sequences due to its fibrous nature (Fig. 5). In some instances, plantar fibroma may show high signal on fluid-sensitive sequences [4].

MRI findings of acute PF tear are complete or partial interruption of the low signal of the PF and signal changes at the site of lesion including high signal on fluid-sensitive sequences and intermediate signal on T1-weighted sequences. High-signal intensity may be an additional finding on fluid-sensitive sequences in the soft tissues surrounding the site of rupture (Fig. 6); this reflects local haemorrhage, inflammation and oedema [2, 66, 67].

Among other lesions of the PF, xanthomas appear as fusiform enlargement of the PF showing heterogeneous signal intensity on both T1- and T2-weighted sequences (Fig. 7) [66]. In the case of foreign body reaction, MRI appearance is variable: low signal intensity on T1-weighted images is frequently noted, while surrounding granulation tissue often has high signal intensity on T2-weighted images [3]. In the case of plantar infection, MR imaging allows identification, localisation and assessment of the extent of the inflammatory process. On MRI, the PF, perifascial soft tissues and adjacent bone show abnormal high signal intensity on fluid-sensitive sequences, low signal intensity on T1-weighted sequences and significant contrast enhancement (Fig. 8) [2, 67].

## Discussion

Based on a systematic review of the last 20 years’ literature and taking advantage of our centres’ experience, this study is able to shed light on key features of PF disorders that can be identified on conventional imaging modalities, such as plain radiography, ultrasound and MRI, thus representing a valuable guide to proper diagnosis of PF disease. Radiographic, sonographic and MRI findings of PF disorders are summarised in Table 1.

**Table 1:** Radiographic, sonographic and MRI features of PF disorders

	Plain radiography	Ultrasound	Magnetic resonance imaging
Plantar fasciitis	PF thickening Narrowed/absent fat pad deep below the PF Cortical changes (sclerosis/lucency and loss of smooth contour) at the PF calcaneal attachment Calcaneal spurs within the PF	PF thickening Loss of fibrillar structure Perifascial fluid collections Calcifications within the PF Hyperemia in the PF/perifascial soft tissues (Doppler imaging) Reduced PF elasticity (elastosonography)	PF thickening Intrasubstance areas of intermediate T1/high T2 signal Oedema in the adjacent soft tissues Bone marrow oedema at the PF calcaneal attachment
Plantar fibromatosis		Iso-hypoechoic, well-demarcated mass No intralesional flow (Doppler imaging)	Lobulated low-signal mass on T1w and T2w images
Tear		Complete/partial interruption of the PF Hypoechoic tissue at the site of rupture	Complete/partial interruption of the PF Intermediate T1/high T2 signal at the site of rupture Oedema in the adjacent soft tissues
Xanthoma		Nodule with speckled pattern	Fusiform enlargement of the PF Heterogeneous T1 and T2 signal
Foreign body	Radiopaque material (e.g., metals)	Echoic material Posterior acoustic shadowing Comet tail reverberation (metals)	Variable signal of the foreign body High T2 signal of the granulation tissue
Plantar infection	Soft tissue swelling Blurring of soft tissue planes Bone osteomyelitic changes	PF thickening Loss of fibrillar structure Perifascial oedema Hyperemia in the PF (Doppler imaging)	Low T1/high T2 signal and contrast enhancement in the PF, perifascial soft tissues and adjacent bone

Plantar fasciitis is the most common disorder of the PF and a frequent cause of heel pain in the general population [22]. Plain radiography, ultrasound and MRI all provide valuable information that aids in the diagnosis. Osborne et al. have demonstrated that PF thickening, abnormalities in the fat pad deep below the PF and bone cortical changes in the calcaneus are radiographic findings of plantar fasciitis [19]. A systematic review of articles published between 2000 and 2012 concerning the role of sonography in plantar fasciitis indicates that it is accurate and reliable [53]. Doppler ultrasound is often normal with plantar fasciitis, but various degrees of hyperaemia may be demonstrated [58–61]. Several studies support the role of elastosonography in patients with plantar fasciitis [62, 63, 68–70], even in symptomatic patients with normal B-mode findings [71]; however, some results are controversial [72] and further investigations are thus needed to clarify the diagnostic value of sonoelastography in plantar fasciitis. Even though there is no significant difference between the accuracy of ultrasound and MRI regarding the measurements of the PF thickness [73], MRI is considered as the most sensitive imaging modality for diagnosing plantar fasciitis [74]. It enables determination of the exact location and extent of the inflammatory alterations within the PF as well as detection of signal changes within adjacent soft tissue or bone marrow [2].

In cases of atypical clinical presentation or where imaging findings do not confirm the presence of plantar fasciitis, differential diagnosis includes other causes of PF disease, such as plantar fibromatosis, trauma and infection, but also disorders arising from structures other than the PF. Ultrasound has several advantages over MRI in the assessment of Ledderhose disease or plantar fibromatosis. Plantar fibromas may be small, thus appearing as small hypointense lesions on MRI, and are difficult to differentiate from the low signal intensity of the PF. Small plantar fibromas are more easily detected on ultrasound because of the contrast between their poorly reflective echotexture and fibrillar appearance of the normal PF. Further, both feet may be examined together using ultrasound. Examining both feet together using MRI reduces the in-plane resolution; in contrast, examining both feet separately is time-consuming if compared with ultrasound [4]. A significant overlap exists between the presentation of plantar fasciitis and that of a traumatic partial tear. In our experience, ultrasound is superior to MRI in differentiating true fibre interruption and tearing from oedema. Confirmation of a complete tear is best achieved by proving widening of the gap between the two ends of PF with dynamic manoeuvres [43]. In cases of complete tear, MRI allows precise estimation of PF retraction with prognostic implications for surgical reparability of the lesion [2]. In cases of infectious fasciitis, MRI provides high anatomic detail and an accurate depiction of the extent of the inflammatory process and adjacent soft tissues, even though artefacts arising from metallic foreign material may be present [40].

Several pathologies involving structures other than the PF may mimic PF disease and should be included in the differential diagnosis. Achilles tendinopathy may present with symptoms that are similar to those of plantar fasciitis. This may be related to the close anatomic connection between the PF and the paratenon of the Achilles tendon [5, 75]. Thus, both the Achilles tendon and PF should be carefully evaluated using ultrasound or MRI. Further, if MRI reveals marked bone marrow oedema at the calcaneal origin of the PF in patients with plantar fasciitis, a concomitant enthesopathy of the Achilles tendon should be suspected [4]. The presence of plantar calcaneal spurs should also be assessed. Spur genesis has traditionally been attributed to chronic traction of the PF and repetitive microtrauma, which in turn lead to periostitis and calcification [3]. The role of vertical compression of the heel in spur formation has recently been hypothesised and related to older age, osteoarthritis and obesity [76]. As stated above, calcaneal spurs are not specific for plantar fasciitis and are often identified in asymptomatic individuals; however, a strong association between spurs and chronic plantar heel pain has been demonstrated, specifically in cases of concurrent fat pad abnormalities [77]. Finally, entrapment of the first branch of the lateral plantar nerve (Baxter’s neuropathy) [43], stress fractures of the calcaneus [78], vascular disease [79] and heel fat pad atrophy and necrosis [2] may present with nonspecific heel pain and represent all differential diagnoses of PF disease.

In conclusion, PF disorders are common causes of heel pain and disability in the general population. Imaging is often required to confirm diagnosis or reveal concomitant injuries. As an inexpensive, quick and dynamic imaging technique that also provides high-resolution depiction of the PF and comparison with the contralateral side, ultrasound should be considered the modality of first choice for assessing PF disorders. Several indirect findings of PF disorders can be detected on conventional radiographs and should be identified even in patients examined for other reasons. MRI can reliably delineate both the soft tissue and bone anatomy of the sole of the foot and enables correct diagnosis of PF disorders, but is expensive and should be regarded as a second-line imaging modality.
